# Implicit bias training can remove bias from subliminal stimuli, restoring choice divergence: A proof-of-concept study

**DOI:** 10.1371/journal.pone.0289313

**Published:** 2023-07-28

**Authors:** Roger Koenig-Robert, Hashim El Omar, Joel Pearson

**Affiliations:** Future Minds Lab, School of Psychology, University of New South Wales, Sydney, Australia; University of Pittsburgh School of Medicine, UNITED STATES

## Abstract

Subliminal information can influence our conscious life. Subliminal stimuli can influence cognitive tasks, while endogenous subliminal neural information can sway decisions before volition. Are decisions inextricably biased towards subliminal information? Or can they diverge away from subliminal biases via training? We report that implicit bias training can remove biases from subliminal sensory primes. We first show that subliminal stimuli biased an imagery-content decision task. Participants (n = 17) had to choose one of two different patterns to subsequently imagine. Subliminal primes significantly biased decisions towards imagining the primed option. Then, we trained participants (n = 7) to choose the non-primed option, via post choice feedback. This training was successful despite participants being unaware of the purpose or structure of the reward schedule. This implicit bias training persisted up to one week later. Our proof-of-concept study indicates that decisions might not always have to be biased towards non-conscious information, but instead can diverge from subliminal primes through training.

## Introduction

Several landmark studies have indicated that our behaviour can be biased or indeed driven by subliminal information. While the extent to which subliminal information affects behaviour is still a contentious topic (see [1] for a review), there is a large body of evidence supporting the influence(s) of subliminal information on behaviour. Examples of such influences are (here named as originally described on the respective articles): implicit attitudes or biases [2], subliminal priming [3], unconscious evidence accumulation in decision making [4, 5], unconscious learning [6, 7], perceptual adaptation from invisible stimuli [8], and voluntary actions and choices triggered by non-conscious brain signals [9–11]. Subliminal information thus can have a wide range of effects on our behaviour contributing to negative behaviour, in the case of unconscious biases [12] to language and motor skills learning in the case of implicit and statistical learning [13] and direct actions in the case of blindsight and agnosia [14, 15].

Research suggests that subliminal information might be processed at variable depths in several brain areas. ‘Invisible objects’ can still be processed through the visual system, especially in dorsal areas [16]—see however [17] for a critical review. Suppressed emotional facial expressions are processed by subcortical structures, in particular the amygdala (for a review see [18], while masked words are semantically processed in the temporal gyrus in the fusiform word area [19].

Subliminal primes have been shown to facilitate or bias behaviour in several cognitive tasks. Subliminal information can accelerate reaction times for semantically related targets [19], improve the accuracy of motion discrimination [4] and speed up reaction times at detecting spoken words after the presentation of subliminal speech [20], among others.

Crucially, research thus far has focused on showing that decisions can lean *towards* subliminal primes. This begs the question of whether choices following subliminal priming are rigidly biased *towards* the subliminal information or could they be trained away, by reversing the bias from the prime. In other words, do we inevitably choose, on average, the same stimuli (or stimuli that share properties with the prime) that were presented subliminally? Another possibility is that we could learn to choose away from subliminal biases, which would mean that such biases and choices are separable. If choices invariably lean *towards* such subliminal primes, this will imply that the subliminal prime triggers a rigid cascade of steps towards a stereotyped behaviour, which determines choosing the primed stimuli. On the other hand, if behaviour can diverge *away* from subliminal biases, this would suggest that the intermediate steps from subliminal perception to behaviour are subject to influence (such as learning) and not always rigidly biased in the same manner.

**Fig 1:**
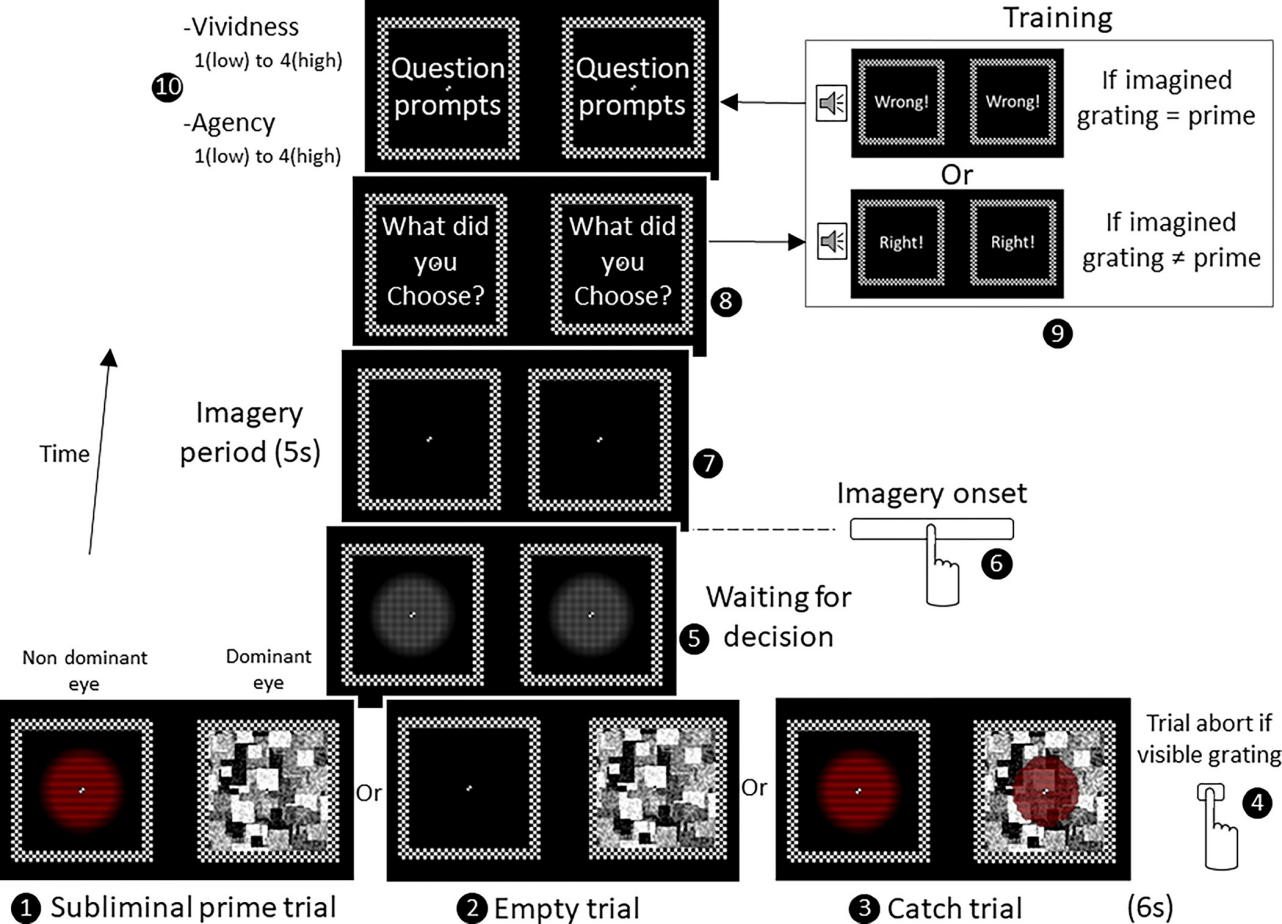
Subliminal priming paradigm. In subliminal prime trials (1), we presented either a red horizontal or a green vertical grating to the non-dominant eye, while presenting a Continuous Flash Suppression (CFS) mask to the dominant eye. In empty trials (2), used as a baseline for vividness and agency ratings, we presented instead an empty screen to the non-dominant eye. In catch trials (3), we presented gratings to both eyes, thus rendering the gratings visible. These trials were used to control for response biases, that is, participants failing to report suppression breaks. Importantly, participants aborted trials at any time when they noticed the primes by pressing a key (4). During the decision-waiting period, a plaid was presented to avoid any afterimages’ influences on the choice (5). After the imagery onset, reported by the participant via a key press (6), participants imagined the grating as vividly as possible for 5s (7). In the no-imagery control experiment, this step was skipped. After the imagery period, participants reported which grating they chose (8) by pressing a key. In the training experiment (9), we gave written and sound feedback on their decisions. Congruent decisions (when the prime and the chosen grating were the same) were considered wrong, while incongruent trials were considered right. Participants were instructed to implicitly learn how to make right decisions. At the end of the trial, participants rated the vividness (how strong the grating visualisation was) and agency (how in control they felt about their decision) from 1 to 4 (10).

The present exploratory study aims to test whether decisions towards a subliminal prime can diverge from it after going through an implicit learning protocol. Specifically, we tested two hypotheses: (1) subliminal primes can bias imagery-content decisions, and (2) participants can be trained to reverse the bias from subliminal primes. We developed a paradigm (Fig 1) in which primes were suppressed from awareness using Continuous Flash Suppression (CFS, [21]). After the presentation of the subliminal prime, participants had to choose one of two gratings and imagine it as vividly as possible. In a subsequent experiment, we gave feedback to a small cohort of participants about their decisions to test whether we could reverse the priming away from the subliminal primes.

## Materials and methods

### Participants

Experimental procedures were approved by the University of New South Wales Human Research Ethics Committee (HREC#: HC17031). All methods in this study were performed in accordance with the guidelines and regulations from the Australian National Statement on Ethical Conduct in Human Research (https://www.nhmrc.gov.au/guidelines-publications/e72). All participants gave written informed consent to participate in the experiments. Participants were recruited during 2017. We tested a total of 29 participants (18 females, ages 18–39) with normal or corrected-to-normal sight for the main experiment. An additional 26 participants (22 retained) were tested in the no-imagery control experiment. Participants’ data were deidentified by assigning them a number so authors could not identify participants after data collection.

### Exclusion criteria

We employed 3 independent selection criteria with the aim of monitor that participants did not consciously perceive the primes. First, we used realistic catch detection trials in which very faint gratings were presented to both eyes to measure participants ability (and willingness) to report subtle suppression breaks that were designed to be as similar as possible to real ones. In catch trials, the luminance of each one of the monocular gratings was lowered to 25% of the intensity of the standard monocular grating. The grating presented to the dominant eye was alpha blended with the CFS Mondrian masks and the other was presented on a black background. This design made catch trials perceptually similar to normal suppression breaks and thus hard to detect in some trials due to monocular suppression [22]. This was done to verify that participants would report even the faintest breakthroughs of the primes. We rejected participants who failed to report at least 80% of catch trials (N = 8). Secondly, we discarded participants who did not report any broken suppression trials (N = 5). While we cannot be sure that some of these participants truly did not experience any suppression breaks, we imposed this criterion in the hope of making sure that participants were not refraining from reporting suppression breaks. Finally, we rejected participants who showed differences in priming between red and green higher than 40% (N = 1). This was done to ensure participants were not biased towards any choice (i.e., choosing red on most trials). Thus, we retained 17 participants who satisfied all these criteria in the main experiment for further analyses. For the no-imagery control experiment, we tested 26 participants. We rejected 4 participants (0 based on criterion 1, 2 based on criterion 2 and 2 others on criterion 3). Despite the use of these criteria, we cannot definitely rule out that participants were able to perceive the primes. While this shortcoming is not unique of our study, there are other controls currently regarded as better suited to detect suppression breaks which were not used in this study (see [23] for a discussion on the subject).

### Power and effect size analyses

**Fig 2:**
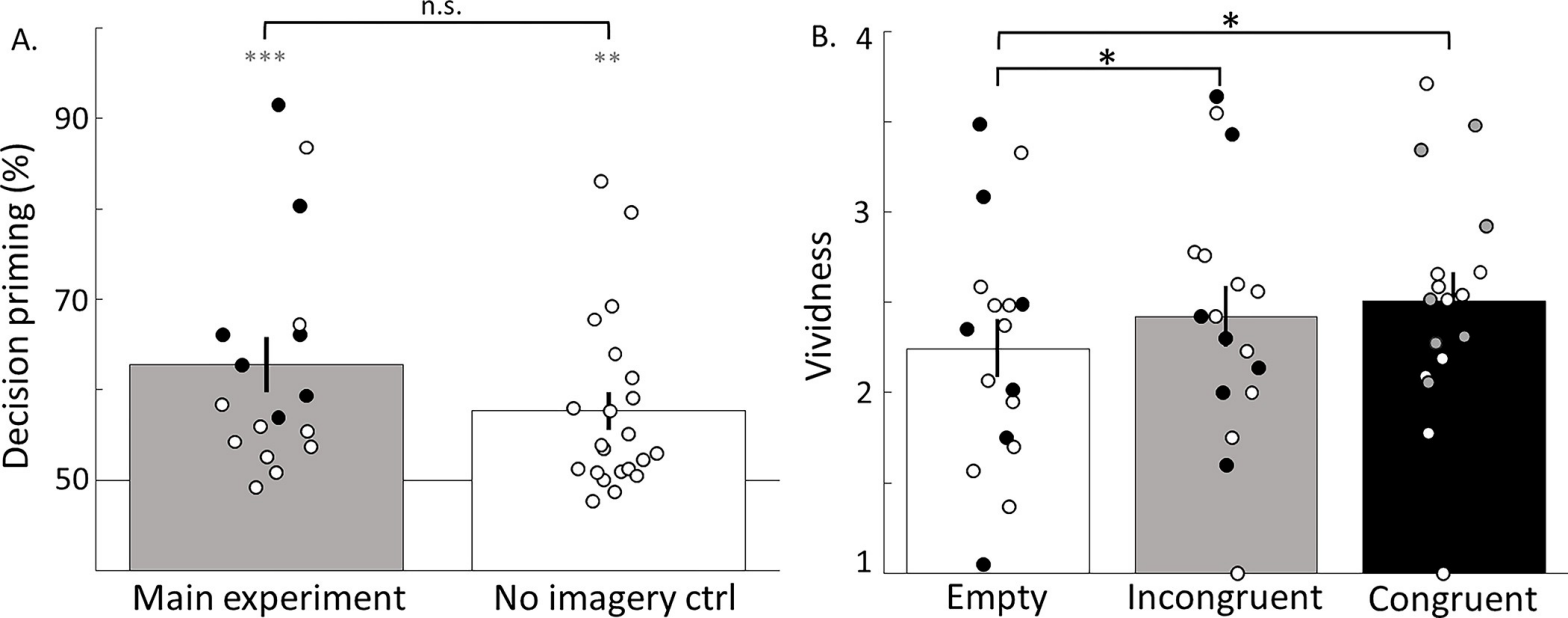
Effects of subliminal gratings. **A. Subliminal priming.** Participants’ decisions to what to imagine were primed by subliminal gratings (grey bar, one-sample t-test against 50%, C.I. 95% = [56.33, 69.21%], t(16) = 4.204, p = 6.72 · 10^−4^). In a control experiment (performed on another cohort of participants n = 22), we removed the imagery component to test to what degree decision priming depended on imagery. In the non-imagery control, priming was lower than in the main experiment (57.7 vs 62.8%) but the difference was not significant (two-sample, one-tailed t-test, C.I. = [-0.88%, Inf], t(37) = 1.44, p = 0.079). **B. Vividness.** Both congruent and incongruent decisions (matching prime and chosen grating and chosen grating different from prime) led to significantly higher vividness compared to empty trials. Congruent decisions vs empty, white vs black bars, paired t-test, C.I. = [0.056, 0.474], t(16) = 2.68, p = 0.0163. Incongruent decisions vs empty, white vs grey bars, paired t-test, C.I. = [0.0035, 0.3545], t(16) = 2.52, p = 0.0227. No significant differences were found between congruent and incongruent decisions (paired t-test, p>0.05). Solid dots represent participants included in the training phase. Error bars represent ±1 SEM.

We performed post-hoc power and effect size analyses using G*Power 3.1 [24] on the main results of the study. For the subliminal priming experiment (Fig 2A), we tested the priming of 17 participants (M = 0.6277, SD = 0.1253) against chance (0.5). The achieved effect size was d = 1.019 and power (1-β) = 0.98, one sample, two-tailed t-test against chance, α error probability = 0.05. For the priming differences post and pre-training, we tested a subset of participants that had significant priming on the main experiment (n = 7). While we acknowledge that this is a rather small sample size, the effect size and power analyses indicate that our results are robust, nonetheless. For the difference between pre-training and training (Fig 4), the effect size was dz = 1.788 (M = 0.298, SD = 0.167) and the power (1-β) = 0.973, two-sample, two-tailed t-test, α error probability = 0.05. Finally, for the difference between pre and post training (Fig 5), the effect size was dz = 1.118 (M = 0.2729, SD = 0.2442) and power (1-β) = 0.694, two-sample, two-tailed t-test, α error probability = 0.05.

### Subliminal prime experiment

The tasks were carried out on a Windows 7 PC running Psychophysics Toolbox 3 [25] in MATLAB (The MathWorks, Inc., Natick, Massachusetts, USA) on a 85Hz Dell Trinitron P1130 CRT monitor 1280x1024 resolution. We presented participants with gratings (red/green, horizontal/vertical, ~7 deg of visual angle, 0.33 monochromatic contrast at its maximum) suppressed using continuous flash suppression (CFS, [21], ~10 deg of visual angle, flickering at 10Hz, square wave). We chose to use coloured gratings instead of grayscale as colour has been shown to promote subliminal priming [26, 27], thus our stimuli had two dimensions that could generate subliminal priming: orientation (horizontal/vertical) and colour (red/green). We started testing perceptual isoluminance of the green and red gratings with the heterochromatic flicker test [28] in which participants were presented with flickering red and green squares (temporal frequency 10Hz) while adjusting the luminance of green against red (constant at 0.33 contrast) until the perceived flickering was minimal. Perceptual isoluminance for green gratings was systematically lower than for red gratings. Subsequently, we tested participants’ eye dominance with the Miles eye dominance test [29] and presented gratings to the non-dominant eye and the dynamic Mondrian patterns (CFS) to the dominant eye to maximize suppression. We used a stereoscope adjusted for each observer until the overlap of each monocular image was achieved while their heads were stabilized using a chinrest. We added a checkerboard pattern square frame around the stimuli (~12 deg of visual angle, ~2 deg width) to aid vergence and thus overlapping of the monocular images. We presented grayscale Mondrian patterns (instead of coloured ones as normally used) and added random noise on them (60% amplitude, zero-centred), which increased the suppression of gratings. The CFS/grating presentation lasted for 6s. To avoid suppression breaks due to abrupt onsets, we linearly increased the opacity of the grating from 0 to a max of 33% (depending on the heterochromatic flicker test) for 2.5s [30]. Importantly, at any moment of the prime presentation as well as in the decision waiting period (see below), participants could abort the trial whenever a prime broke suppression. We used empty trials (40%), where no gratings were presented as baseline for vividness and agency ratings. We also presented catch trials (20%) in which gratings were shown to both eyes to control for decision biases on reporting suppression breaks. We used realistic catch detection trials in which very faint gratings were presented to both eyes. The intensity of each monocularly presented grating was 25% of the gratings presented in a standard trial. This design was used to replicate real suppression breaks, because break-through gratings can be difficult to detect. Thus, we expected that catch trials could be missed in some trials. A total of 150 trials per participant were tested divided in 5 blocks of 30 trials each, with trials from different conditions pseudorandomized within blocks. After the prime presentation, participants had to decide which grating to imagine. During this waiting period, we presented grayscale plaids matching the position of the primes to mask any after images of the prime that could influence the decision. After participants decided which grating to imagine, indicated by pressing space bar (independently of which one of the gratings was chosen), they imagined it as vividly as possible for 5 seconds. After the imagery period, participants communicated their choice by pressing different keys on a computer keyboard. After this, participants were required to rate the strength of their visualizations or vividness on a scale from 1 to 4. Finally, they rated on the same scale the sense of agency felt while deciding which grating to imagine.

### Prime and decision dissociation training experiment

We implicitly trained participants to switch their decisions away from the subliminal prime. For this experiment, we selected a subset of the retained participants from the main experiment. We selected participants with priming significantly above chance across the mean priming values within each of the 8 experimental blocks (one sample, one-tailed t-test against 50% p<0.05). See the Discussion section for the rationale behind this choice. We thus selected 7 participants who showed significant above chance priming and trained them in 3 sessions performed on consecutive days plus a post-training session to test the persistence of the training effect a week after the training. Importantly, the selection criterion was defined, and participants were selected *before the training session*, so no participants were discarded after the training, nor we stopped testing participants after obtaining significant results.

**Fig 3:**
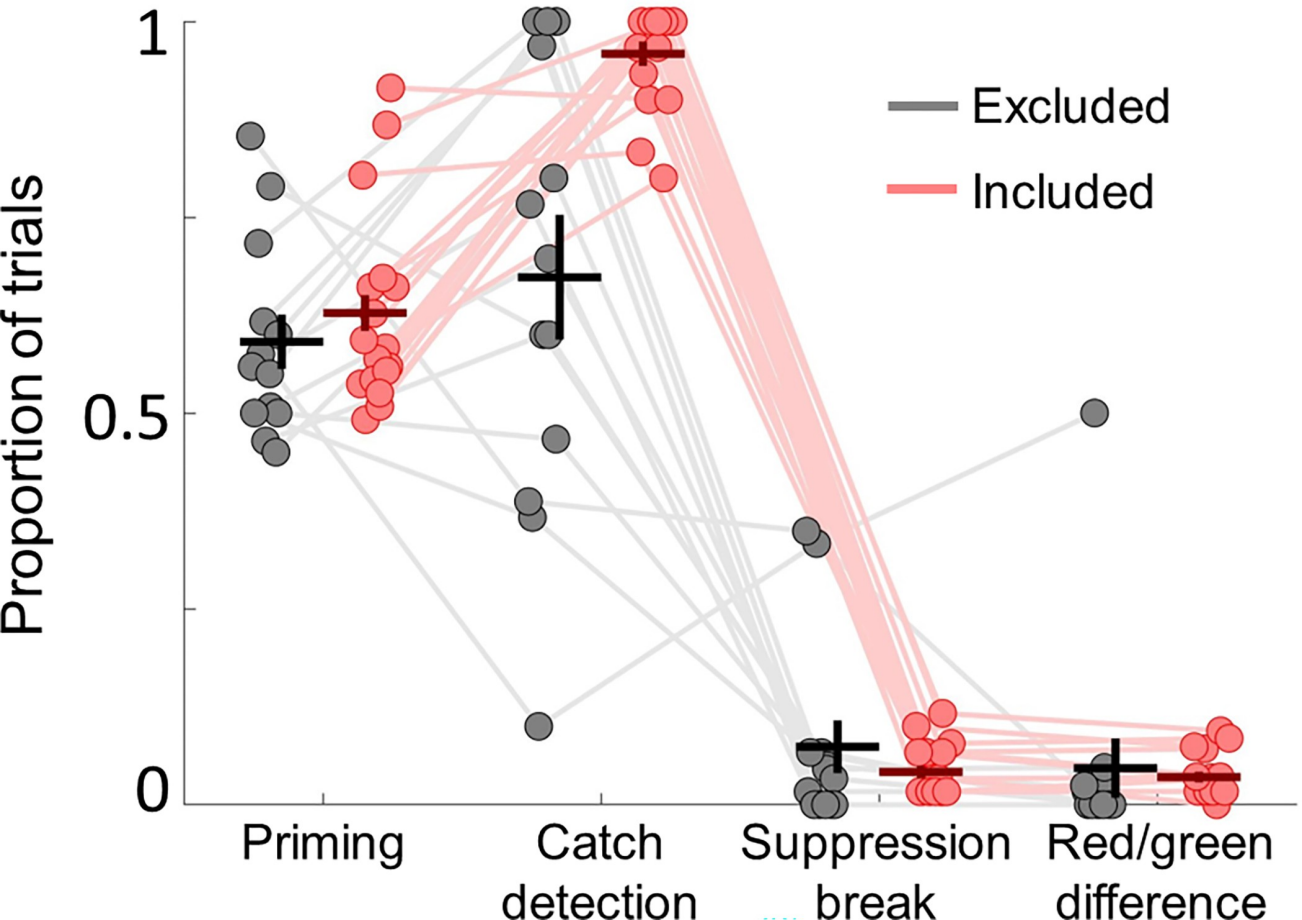
Summary of the exclusion criteria measures used to ascertain subliminal priming. We used 3 stringent exclusion criteria to ascertain the reliability of participants’ reports on the priming task: catch detection, suppression breaks and priming differences. We excluded participants failing to report <80% of the catch trials (N = 8). Mean catch detection ratios for excluded and included participants were 0.673 (±0.079 SEM) and 0.959 (±0.016 SEM) respectively and were significantly different (p = 4.3·10^−4^, C.I. = [-0.4321–0.1389], t(28) = -3.9896, two sample t-test). We also excluded participants who did not report suppression breaks (N = 5). Mean suppression break ratios for excluded and included participants 0.074 (±0.034 SEM) and 0.042 (±0.008 SEM), respectively, but were not significantly different (p = 0.305). Lastly, we excluded participants that had differences in priming between red and green gratings larger than 40% (N = 1) as this imbalance could indicate that participants’ choices are biased towards one option. Differences in red/green priming between excluded and included participants were 0.047 (±0.038 SEM) and 0.035 (±0.007 SEM), respectively, but were not significantly different (p = 0.74). Mean priming ratios for excluded and included participants were 0.591 (±0.035 SEM) and 0.628 (±0.023 SEM) respectively but were not significantly different (p = 0.432). Circles represent single participants and thin lines link single participant data. Thick horizontal and vertical lines represent group mean and ±1 SEM respectively. Black and red represent excluded and included participants respectively.

**Fig 4:**
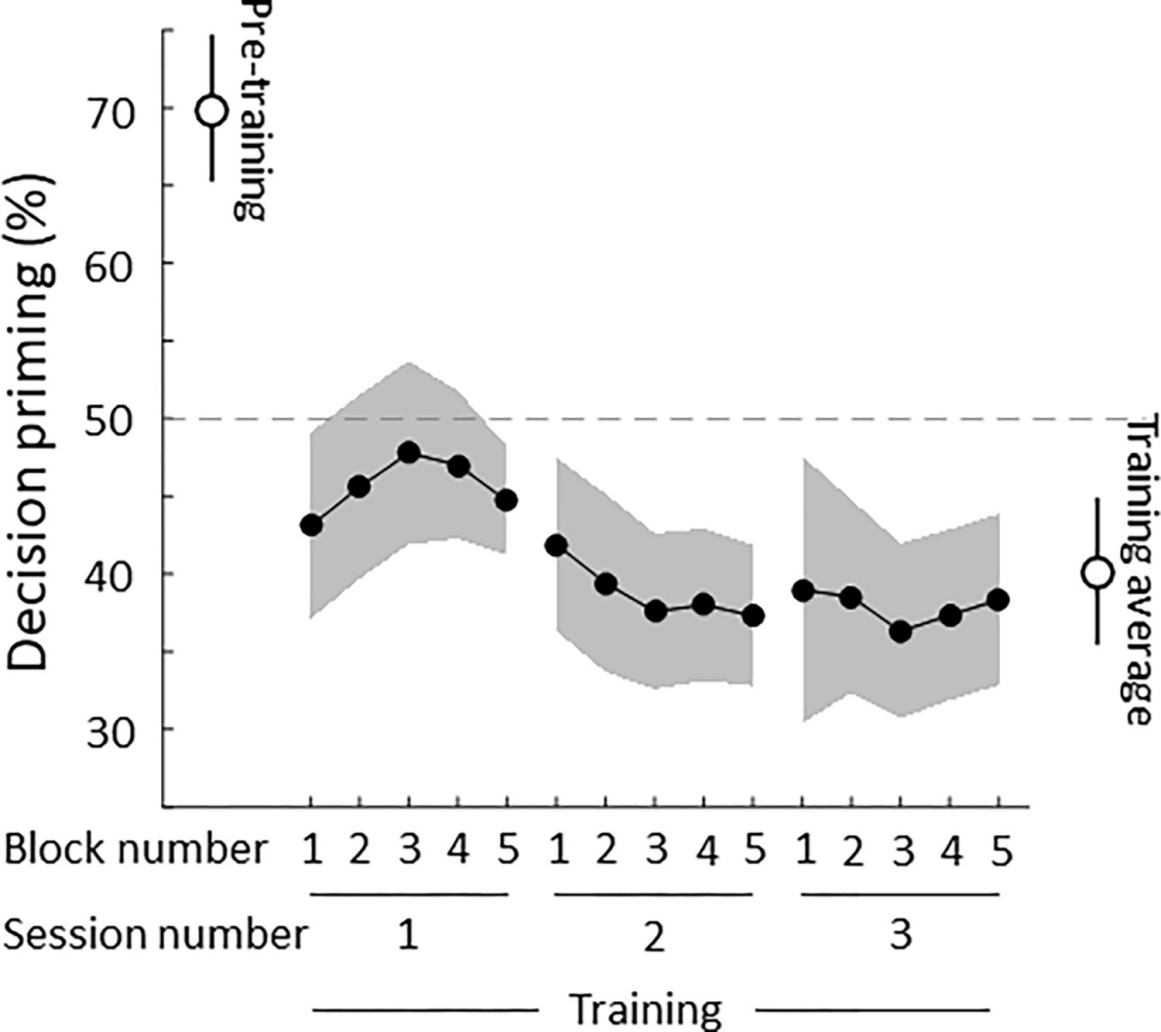
Subliminal primes and decisions are dissociable through implicit training. We trained a subset of participants who showed significant priming on the decision task (N = 7, see Materials and Methods for selection criteria) to implicitly learn to choose an incongruent grating with respect to the prime. This training was performed in 3 sessions of 5 blocks each. Pre-training priming on the participants’ subset was 69.89% (one sample t-test against 50%, C.I. = [58.48, 81.3%], t(6) = 4.265, p = 0.0053). Training priming levels were significantly different from pre-training (paired t-test, p = 0.0104, 0.0082, 0.0082 for sessions 1 to 3, FDR corrected, q = 0.05), but only significantly lower than baseline in session 2 (one sample, one-tailed t-test, p = 0.0901, 0.0473, 0.0595, FDR corrected, q = 0.05). On average, implicit training resulted in participants switching their decisions towards the incongruent grating significantly below chance level (average training priming = 40.11%, one-sample t-test against 50%, C.I. = [31.92, 48.3%], t(6) = -2.95, p = 0.0255). Error bars/shaded area represent ±1 SEM.

During the training sessions, performed weeks after the main experiment, participants were presented with the same paradigm as in the main experiment except for the following changes. After participants reported the chosen grating, written and audio feedback was given. For trials where participants chose the same grating as the prime (congruent trials), we presented the word “Wrong!” and played a beep sound. Inversely, in trials where participants did not choose the priming grating (incongruent trials), we presented the word “Right!” and played a bell sound. In catch and empty trials, decisions were labelled as right no matter the grating chosen. We asked participants to try to get as many right trials as possible. The instructions given to the participants were: “you will be presented with the same paradigm as in the previous experiment you performed weeks ago. Each trial will start with flashing stimuli followed by a decision imagery task as previously. As in the previous experiment, if you happen to detect a grating or red/green colour during the flashing stimuli, you must report it. This time, however, at the end of the trial there will be feedback: ‘right’ and ‘wrong’. Try to get as many ‘right’ feedback even if you don’t know how to do it". In post-experiment interviews, participants reported not knowing how to perform the task, however, priming ratios revealed that they could implicitly learn to choose the right grating (incongruent, Figs 3 and 4).

### No-imagery decision control

We devised a control experiment to test to which degree the task requirement of producing a mental image after the decision influenced the subliminal priming. We thus presented to an independent set of participants the same task as described in the subliminal prime experiment, except for that there was no imagery period nor vividness rating.

### Priming analysis

We calculated priming by dividing the number of trials where the choice was congruent with the prime by the total number of trials that were not aborted. These ratios were subsequently multiplied by 100 to denote percentages. Statistical analyses were thus focused on testing that the amount of priming was either different from chance level, i.e., 50% or comparing priming levels between different conditions/experiments.

## Results

### Paradigm

The paradigm consisted of an imagery-content decision task following the presentation of a subliminal prime suppressed using Continuous Flash Suppression: CFS [21]. Details of the paradigm and stimuli generation are available in the Materials and Methods section as well as in Fig 1‘s caption. Importantly, participants were instructed to abort trials at any time when the gratings (primes) became visible by pressing a key (Fig 1.4). We included control trials to test for response bias (catch trials, Fig 1.3) and as a baseline for vividness and agency ratings (empty trials, Fig 1.2). For a schematic of what each trial type looked like, see S1 Fig in S1 File.

### Subliminal gratings prime decisions about the contents of future imagery

We first tested whether the presentation of subliminal gratings biased participants’ choices towards subliminal stimuli. Our results indeed showed significant priming by the subliminal gratings on the subsequent imagery decision (mean 62.8% ±3.04 SEM, Fig 2A, grey bar, one-sample t-test against 50%, C.I. = [56.33, 69.21%], t(16) = 4.204, p = 6.72 · 10^−4^, solid dots represent participants included in the training phase). We devised a control experiment to test to what degree the decision priming depended on imagery. The no-imagery control experiment was identical to the main experiment with the exception that there was no imagery period, nor vividness rating question (Fig 1). Average priming in the control condition was again significantly above chance (mean 57.65% ±2.07 SEM, Fig 2A white bar, one-sample t-test against 50%, C.I. = [53.35, 61.95%], t(21) = 3.6986, p = 0.0013). While the control priming was slightly lower than in the main condition (5.15% difference) this difference was not significant (p = 0.079, two-sample, one-tailed t-test). This result suggests that the decision priming is essentially independent of the subsequent imagery.

We then compared the vividness ratings between primed and empty trials. We used empty trials (where no prime was presented) to measure the baseline vividness levels. This analysis revealed that, in primed trials, when the prime and the choice were congruent, vividness (the subjective imagery strength) was higher (Fig 2B, white vs black bars, paired two-tailed t-test, C.I. = [0.056, 0.474], t(16) = 2.68, p = 0.0163), than when no prime was presented (empty trial). The vividness on incongruent decisions, while lower on average when compared to congruent decisions, was also significantly higher than the empty condition (Fig 2B, white vs grey bars, paired t-test, C.I. = [0.0035, 0.3545], t(16) = 2.52, p = 0.0227). No significant differences were found between vividness ratings for congruent and incongruent decisions (Fig 2B). This suggests that, in this particular paradigm, showing subliminal primes boosted vividness regardless of the congruency with the imagined item.

Analyses of agency ratings did not show significant differences among the different conditions of the main experiment: empty (baseline), primed and non-primed trials (S2 Fig in S1 File). The lack of a difference in ratings of agency suggests that participants were largely unaware of the influence of the subliminal primes on their decisions, or at least they were not aware of any change in their agency.

We used 3 exclusion criteria to strengthen the reliability of reports on the subliminal priming task: catch detection, suppression breaks and priming differences. Participants not satisfying any of these 3 criteria were excluded from further consideration (Fig 2 open dots and Fig 3 in grey). First, we used realistic catch detection trials in which very faint gratings were presented to both eyes (i.e., not easily detectable, see Methods for details) to measure participant’s ability (and willingness) to report subtle suppression breaks. As catch trials were designed to replicate real suppression breaks, they were expected not to be reported in some trials. We, therefore, excluded participants that failed to report at least 80% of the catch trials (N = 8). Mean catch detection ratios for excluded and included participants were 0.673 (±0.079 SEM) and 0.959 (±0.016 SEM) respectively and were significantly different (p = 4.32·10^−4^, C.I. = [-0.4321–0.1389], t(28) = -3.9896, two sample t-test). We also excluded participants who did not report any suppression breaks (N = 5). Mean suppression break ratios for excluded and included participants 0.074 (±0.034 SEM) and 0.042 (±0.008 SEM), respectively, but were not significantly different (p = 0.305). Lastly, we excluded participants (N = 1) that had differences in priming between red and green gratings larger than 40% (for example 80% red) as this imbalance could indicate that participants’ choices would be biased towards one particular option (e.g., choosing red). Differences in priming between excluded and included participants were 0.047 (±0.038 SEM) and 0.035 (±0.007 SEM), respectively (see Fig 3), but these were not significantly different (p = 0.74). Mean priming ratios for excluded and included participants were 0.591 (±0.035 SEM) and 0.628 (±0.023 SEM) respectively but were not significantly different (p = 0.432), indicating that priming was not explained by differences in the exclusion variables.

We performed additional tests to ascertain if the inclusion criteria for suppression break ratios could explain the priming results. We thus analysed whether priming scores were correlated with suppression breaks. Our analyses did not detect significant correlations (p>0.1), although there was a slight positive trend, between suppression break ratios and priming (S3 Fig in S1 File), thus suggesting that partial failure at reporting suppression breaks is not enough to explain priming results.

### Implicit training makes decisions diverge from subliminal primes

To test whether decisions are bound to subliminal primes, we trained a subset of participants who had significant above chance priming across blocks (N = 7, see Materials and Methods for details in the selection criteria details) to choose the grating that was not primed. We used the same paradigm as in the main experiment, except that we included a feedback section after they reported their decision. For primed trials, where the decision matched the subliminal grating, we presented the prompt “Wrong!” accompanied with a buzz sound. Inversely, when participants chose the grating that was not presented as a prime, we presented the prompt “Right!” accompanied with a bell sound. On empty and catch trials, the “Right!” prompt was shown independent of the answer. Systematic post-experimental verbal interviews after each session revealed that 100% of participants (7 out of 7) responded “no” to the question “Were you aware of how to perform the task?”. Despite this, participants learnt quickly how to make their choices diverge from the subliminal primes. We trained participants in 3 sessions of 5 blocks each (Fig 4). Pre-training priming on the participants’ subset was 69.89% ±4.66 SEM (one sample t-test against 50%, C.I. = [58.48, 81.3%], t(6) = 4.265, p = 0.0053). Training priming levels were significantly different from pre-training (paired t-test, p = 0.0104, 0.0082, 0.0082 for sessions 1 to 3, FDR corrected for multi-comparisons, q = 0.05), but only significantly lower than chance level (i.e., 50%) in session 2 (one sample t-test, p = 0.0901, 0.0473, 0.0595, FDR corrected, q = 0.05). On average, implicit training resulted in participants switching their decisions towards the incongruent grating significantly below chance level (average training priming = 40.11% ±4.75 SEM, one-sample t-test against 50%, C.I. = [31.92, 48.3%], t(6) = -2.95, p = 0.0255).

### Implicit training effects persist over time

**Fig 5:**
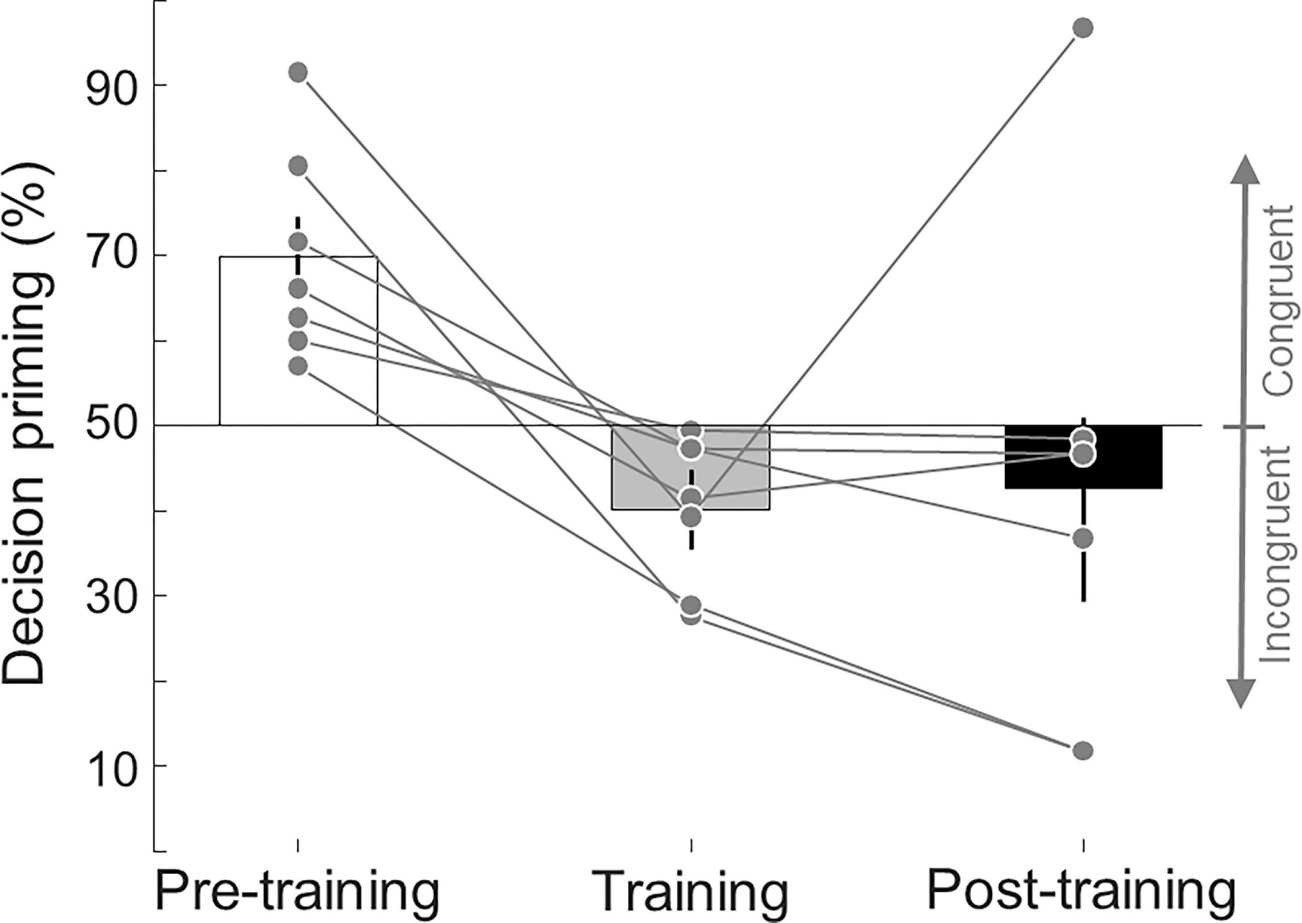
Training effect remained one week after in most participants. All participants shifted their decisions against the subliminal prime during the training phase (lines represent individual participants). After one week, all but one participant (6 out of 7, 85.7%) kept their decision responses incongruent to the subliminal prime. Post-training decision priming was significantly lower than pre-training levels (paired t-test, C.I. = [-49.87, -4.71%], t(6) = -2.96, p = 0.025), while training and post-training decision priming levels were not significantly different (paired t-test, C.I. = [-21.23, 26.21%], t(6) = 0.257, p = 0.806). Error bars represent ±1 SEM.

Finally, we investigated whether the effect of training was robust over time. We thus tested the same trained participants one week later. We used the same paradigm as in the main experiment, that is, *without* any feedback. During the training phase, all participants switched their decisions towards the grating that was not shown as prime, in other words, they chose to make incongruent decisions (Fig 5). After one week, all but one participant (6 out of 7, 85.7%) kept their decision responses incongruent with the subliminal prime. Post-training decision priming (42.6% ±10.84 SEM) was significantly lower than pre-training levels (paired t-test, C.I. = [-49.87, -4.71%], t(6) = -2.96, p = 0.025), while training and post-training decision priming levels were not significantly different (paired t-test, C.I. = [-21.23, 26.21%], t(6) = 0.257, p = 0.806).

Interestingly, agency ratings fell modestly but significantly from pre-training to post-training levels (S4 Fig in S1 File, C.I. = [0.0335, Inf], t(6) = 2.053, p = 0.043). This suggests that participants may have become more aware of the influence of subliminal information on their choices, even if they reported not being aware of the subliminal primes or the purpose of the task. On the other hand, we found no significant changes in vividness across the pre-training, training, and post-training experiments (S5 Fig in S1 File). Participants’ catch trial and break suppression rates were not significantly different along training periods (S6 Fig in S1 File), ruling out major changes in strategies acquired during the training.

These results suggest that the associations made between the subliminal primes and decisions during the training can persist over time, even in the absence of reinforcement.

## Discussion

We developed a paradigm to test whether subliminal primes can bias decisions about the contents of future imagery. Our results indicate that participants’ choices were biased towards the subliminal prime. It is important to highlight that there is still debate surrounding the nature of non-conscious perception (for a review see [1]), and there have been a number of studies questioning the use of CFS to study non-conscious perception based on methodological grounds [23, 31–33]. We used controls to minimize concerns about participants performing the task incorrectly (Fig 3). First, we used realistic looking catch trials where very faint (25% of the subliminal prime contrast) gratings presented to both eyes. We excluded participants failing to report at least 80% of catch trials. Additionally, we also excluded participants failing to report any suppression breaks during normal trials. While some of these participants could have genuinely experienced no suppression breaks, we used this criterion to filter out potential participants that could be systematically not reporting catch trials. Further, we excluded participants that were disproportionally primed by one of the coloured gratings as this can be evidence of a bias on their choices (see Methods for inclusion criteria details). Finally, we verified that, for participants passing the inclusion criteria, the rate of suppression breaks was not significantly correlated with priming. This analysis suggests that priming is unlikely to be a result of partial reports of suppression breaks (S3 Fig in S1 File). To the best of our knowledge, these selection criteria have not used in combination on previous studies, and due to the stringent nature of these criteria, we ended discarding an important number of participants (12 out 29 or 41%). While this is not unique to our study (see for example [34]) we understand the need to replicate our results to assess their full impact. As noted before, however, priming on the discarded participants was not significantly different from priming on the included participants (Fig 3), indicating that our results cannot be explained by our selection criteria. It is also important to emphasize that despite our efforts to ascertain that participants were unable to see the gratings, we cannot definitively rule out this possibility. As epistemological and methodological views on non-conscious perception continually evolve to address the shortcomings of previous studies (e.g., [23]), it is up to future research to replicate these results with the latest methods to date to dissociate non-conscious perception.

In the follow up experiment, we tested a subset of participants (n = 7) and found that choices could diverge from subliminal prime after implicit training. We gave participants feedback on their choices, rewarding divergence from the primes while penalising congruent decisions. Despite participants systematically reporting being unaware of how to perform adequately at the task (which was evaluated by systematic post-experimental verbal interviews), they quickly learnt to diverge from the primes, on average switching their decisions from the first session. Participants implicitly learnt to choose the grating that was not primed, leading to a significant decrease in choice priming compared to pre-training levels. We acknowledge that, due to the selection criterion (within-participant priming significantly above chance), we ended up with a rather small sample size (N = 7). Calculating significant priming within participants has not been done before in the literature, due to the variability across blocks/trials (in this respect, our sample was comparable to previous studies) and also because there has been no need to draw conclusions at the participant level. Instead, previous studies have used average within participant priming and calculated significance across participants [35, 36]. We however reasoned that using within-participant significant priming as a selection criterion would allow us to highlight changes produced by the implicit training at the participant level while maximizing the sensitivity of our experimental procedure by avoiding floor effects (i.e., if a participant’s priming was only barely above chance level, it would have been hard to detect a change after training if the effect size was small). While in hindsight this selection criterion might have been too stringent (another option could have been selecting participants with average priming above 50%, no matter whether significant or not), we chose it because we could not anticipate the effect size, which ended up being rather large, with most participants reversing their choices (i.e., priming below 50%). Therefore, a larger sample study will be needed to reveal the full scope of these findings, despite that our analyses showed remarkable large effect sizes and satisfactory power values (see Methods for details).

Our results suggest that decisions are not determined to be congruent with subliminal representations but can diverge through learning. This capacity of learning can seem surprising given that non-conscious behaviour has been classically regarded as rigid and stereotyped [37–39]. However, recent studies have shown rather complex learning in the absence of awareness such as fear conditioning [40], sequence regularity learning [41] and instrumental conditioning [42], see however [43] for evidence against unconscious instrumental conditioning. Our results can be seen as another case of instrumental learning in the absence of awareness, this time implicit learning to diverge from subliminal primes.

Implicit training effects persisted after a week despite the lack of reinforcement. This is consistent with reports on the robustness and long-lasting effects of the non-conscious learning [44, 45]. This result suggests that the ability to dissociate subliminal information from choices can last long enough to change behaviour in a meaningful way. This can be of potential interest for the treatment of mental conditions in which non-conscious material affects behaviour, such as in depression [46–48].

Participants’ agency felt modestly but significantly when comparing pre-training with post-training (but not when comparing pre-training vs training nor training vs post-training), which could suggest that participants may have become more aware about the influence of subliminal information on their choices. Participants, however, consistently reported not being aware of the subliminal primes nor the purpose of the task. In addition, participants’ catch-trials detection remained high and didn’t significantly change across the training stages, indicating that participants were reporting suppression breaks appropriately. Further, the instruction given to the participants did not hint at the relationship to-be-learned (i.e., choose the grating opposite to the prime) and was basically “try to get more right feedback” (see Methods for details on the instructions), suggesting that participants learnt the new association between the subliminal prime and their decision outside of awareness, thus learning implicitly [49]. As for the agency reports falling through the training process, this is consistent with studies reporting dissociations between agency ratings and non-conscious information [50, 51]. That is, participants have been shown to rate their agency lower while not being aware of external influences on their behaviour.

In summary, this proof-of-concept study opens new possibilities in the study of the effects of subliminal information on decision-making.

## Supporting information

S1 File Supporting information figures. File including supporting information figures S1 to S6 Figs.
(PDF)
