# Supplementary material for: Implicit bias training can remove bias from subliminal stimuli, restoring choice divergence: A proof-of-concept study
Source: PLoS One. 2023 Jul 28;18(7):e0289313. doi: 10.1371/journal.pone.0289313 (PMC10381032; doi:10.1371/journal.pone.0289313)
Supplement: S1 File — File including supporting information figures S1 to S6 Figs. (PDF) [file pone.0289313.s001.pdf]

Supporting Information

Implicit bias training can remove bias from subliminal stimuli, restoring choice divergence: A proof-of-concept study.

Roger Koenig-Robert, Hashim El Omar and Joel Pearson

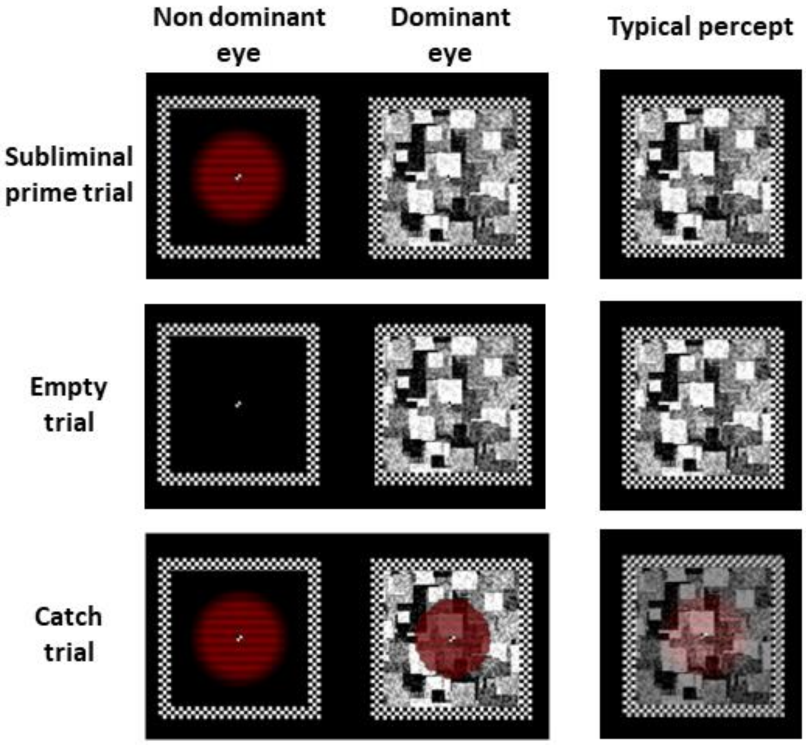

S1 Fig. Schematics of typical percept for each trial type. The typical percept in Subliminal prime trials was the flashing Mondrian patterns. In case the grating broke suppression, participants were instructed to report the suppression by pressing a key. In empty trials, the typical percept was the Mondrian patterns. These trials were used to calculate baseline agency and vividness. Typical percept in catch trials was a very faint grating (strength of the grating exaggerated on the Fig for display purposes). These faint catch trials were designed to look like real suppression trials and expected to be missed on some trials.

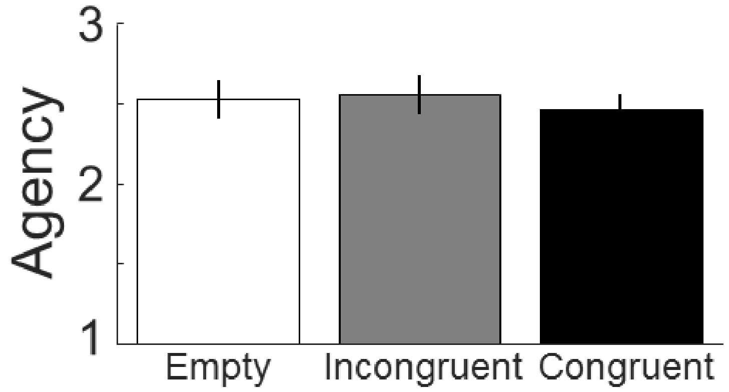

S2 Fig. Agency for different conditions for the main experiment (N=17). No differences in agency were seen among the different conditions (p>0.05, two-tailed paired t-test). Similar results were found considering the contingent chosen for training (N=7).

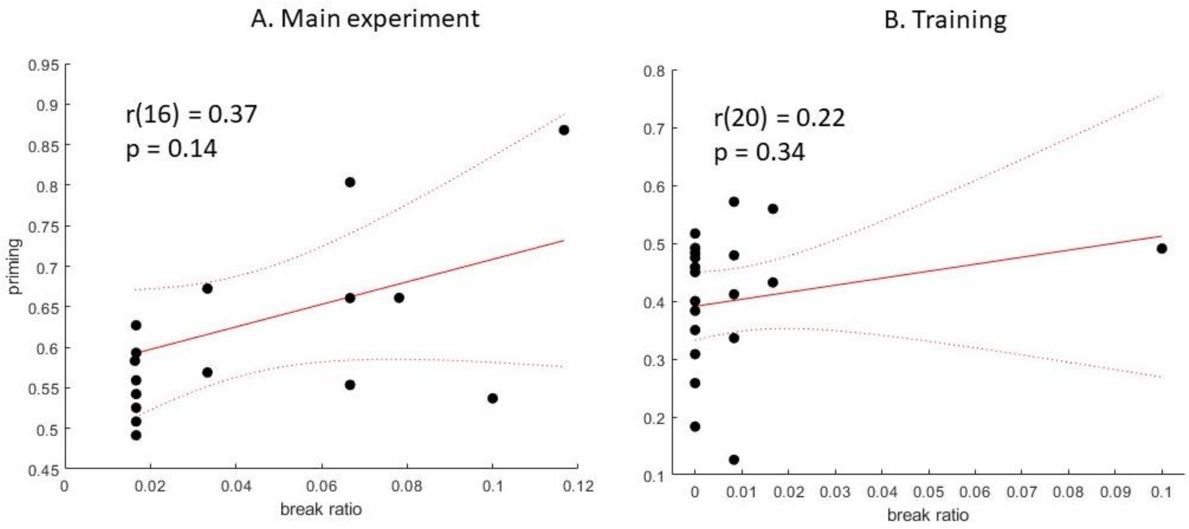

S3 Fig. Priming as a function of suppression break ratio. We found no significant correlation between suppression break ratios and priming. **A.** Main experiment, r(16) = 0.3733, p = 0.1413. **B.** Training. To ensure statistical power, linear correlation between suppression break ratios and priming was calculated taking each session (n=3) and participant (n=7) independently, thus 21 data points were feed into the model. The results showed no significant correlation r(20) = 0.2193, p = 0.3396. Correlation using only mean values per each subject (n=7 data points) did not show significant results either: r(6) = -0.03, p = 0.9492. Dots represent single subject data in A and single session data in B. Solid red line represents linear fit. Dashed red line represents 95% confidence intervals.

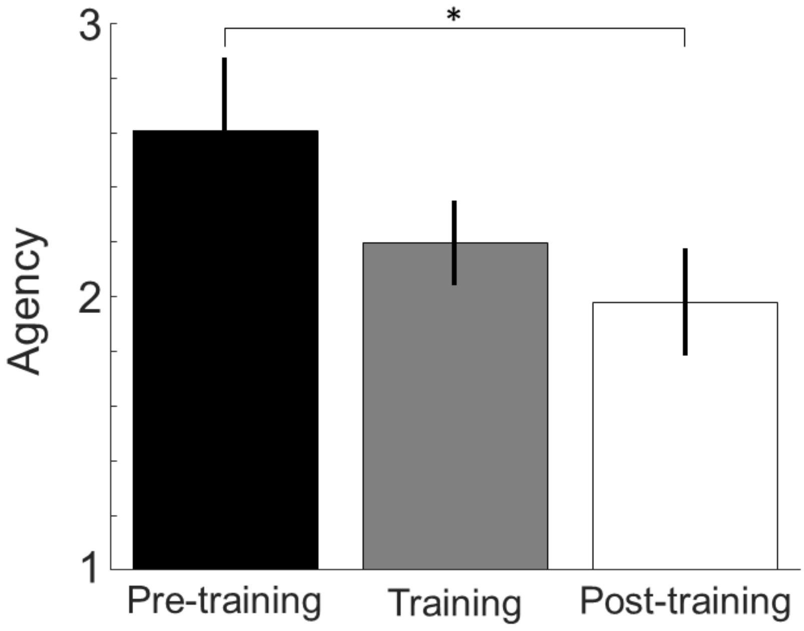

S4 Fig. Agency across training on congruent trials. Post-training agency fell significantly compared to pre-training levels (one-tailed paired t-test p = 0.043, C.I. = [0.0335, Inf], t(6) = 2.053, non-corrected for multicomparisons). While average agency was lower in during training compared to pre-training levels, this effect was not significant (one-tailed paired t-test p = 0.102, C.I. = [-.149, Inf], t(6) = 1.429).

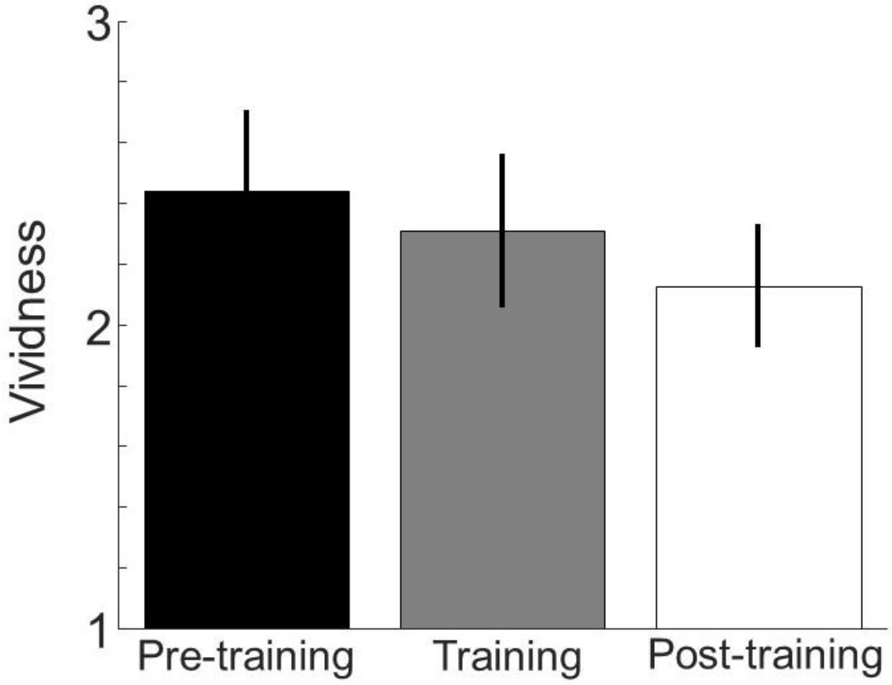

S5 Fig. Vividness across training on congruent decisions. We found no significant differences in vividness across experiments (Pre vs training, p=0.74; pre vs post training p = 0.39, two-tailed paired t-test).

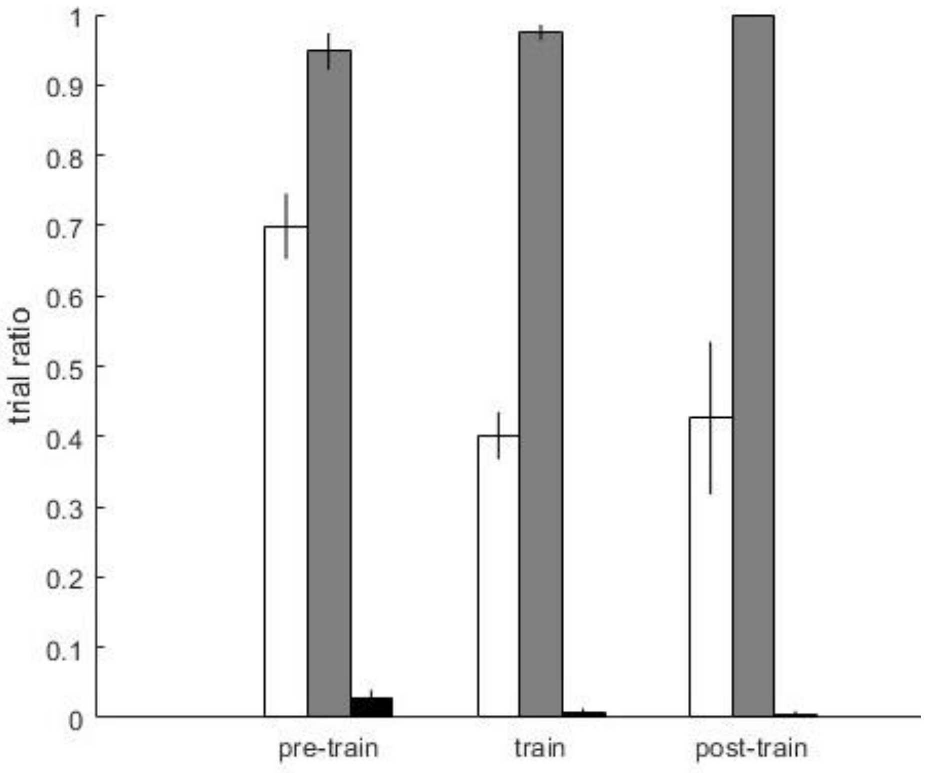

S6 Fig. Priming (white), catch-detection (grey) and suppression breaks (black) across training. While priming values were significantly different across the pre-training, training and post-training groups, $F(2, 18) = 5.44$, $p = 0.0142$, one-way ANOVA, we found no differences in catch detection rates, $F(2, 18) = 2.57$, $p = 0.1046$, nor suppression breaks, $F(2, 18) = 3.05$, $p = 0.0724$.
